# Androgen Receptor/AP-1 Activates *UGT2B15* Transcription to Promote Esophageal Squamous Cell Carcinoma Invasion

**DOI:** 10.3390/cancers15245719

**Published:** 2023-12-06

**Authors:** Jiahui Cai, Furong Huang, Wenyan Gao, Tongyang Gong, Hongyan Chen, Zhihua Liu

**Affiliations:** 1 State Key Laboratory of Molecular Oncology, National Cancer Center, National Clinical Research Center for Cancer, Cancer Hospital, Chinese Academy of Medical Sciences and Peking Union Medical College, Beijing 100021, Chinamaimm111@163.com (F.H.); gao_wen_yan@163.com (W.G.); gongty09@126.com (T.G.); 2 Key Laboratory of Cancer and Microbiome, National Cancer Center, National Clinical Research Center for Cancer, Cancer Hospital, Chinese Academy of Medical Sciences and Peking Union Medical College, Beijing 100021, China

**Keywords:** esophageal squamous cell carcinoma, androgen receptor, AP-1, UGT2B15, invasion, metastasis

## Abstract

**Simple Summary:**

Emerging evidence suggests that AR is involved in the growth of esophageal squamous cell carcinoma. However, how AR exerts its functions in the invasion of esophageal squamous cell carcinoma remains unknown. In this study, by analyzing our previous AR cistromes and androgen-regulated transcriptomes, we find that AR upregulates the expression of UGT2B15 in esophageal cancer cells. We further find that the transcription factor AP-1 governs AR-induced transcription. Importantly, genetic or pharmacological inhibition of AP-1 relieves AR-mediated UGT2B15 activation, leading to esophageal cancer cell invasion inhibition. Our findings reveal the molecular mechanisms of AR in esophageal cancer invasion and identify the AP1-directed AR transcriptional activation of UGT2B15 as a potential therapeutic target for esophageal cancer metastasis.

**Abstract:**

Esophageal squamous cell carcinoma (ESCC) is an aggressive epithelial malignancy with poor prognosis. Interestingly, ESCC is strongly characterized by a male-predominant propensity. Our previous study showed that androgen receptor (AR) orchestrated a transcriptional repression program to promote ESCC growth, but it remains unclear whether AR can also activate oncogenic signaling during ESCC progression. In this study, by analyzing our previous AR cistromes and androgen-regulated transcriptomes, we identified uridine diphosphate glucuronosyltransferase family 2 member B15 (UGT2B15) as a bona fide target gene of AR. Mechanistically, AP-1 cofactors played important and collaborative roles in AR-mediated *UGT2B15* upregulation. Functional studies have revealed that UGT2B15 promoted invasiveness in vitro and lymph node metastasis in vivo. UGT2B15 was partially responsible for the AR-induced invasive phenotype in ESCC cells. Importantly, simultaneous blocking of AP-1 and AR resulted in stronger inhibition of cell invasiveness compared to inhibiting AP-1 or AR alone. In conclusion, our study reveals the molecular mechanisms underlying the AR-driven ESCC invasion and suggests that the AR/AP1/UGT2B15 transcriptional axis can be potentially targeted in suppressing metastasis in male ESCC patients.

## 1. Introduction

Esophageal squamous cell carcinoma (ESCC) accounts for nearly 90% of all esophageal cancer cases and is the most common histological subtype in China [1]. ESCC ranks sixth among the leading causes of cancer-related death with an overall 5-year survival rate of about 20% [2,3]. Interestingly, ESCC is characterized by a strong male-predominant propensity, with two- to three-fold higher incidence and mortality rates in male compared with female individuals [2]. The male-predominant propensity of ESCC could be attributed mostly to the sex differences in exposure to major risk factors including tobacco smoking and alcohol consumption [1]. Nevertheless, studies over the past several decades have suggested that sex hormone signaling (i.e., estrogen and androgen) was associated with the survival of ESCC patients [4,5] and exerted either oncogenic or tumor suppressive functions depending on specific receptor subtypes [6,7], implying that sex hormones may contribute to the gender disparity of ESCC.

While several studies have revealed the crucial role of androgen receptor (AR) signaling in male-dominant ESCC [8,9], our previous study has uncovered the oncogenic functions of AR in driving ESCC progression [10]. Despite liganded AR mainly repressing the transcription of tumor suppressor genes, androgen treatment also induces transcriptional activation in AR-expressing KYSE410 cells, which is similar to the dual functions of AR-mediated gene regulation in prostate cancer [11]. However, it is unknown how AR upregulates the transcription of target genes and whether these genes play oncogenic functions in ESCC cells.

It has been well-established that genome-wide AR signaling relied on the combinatorial recruitment of AR, DNA-binding collaborating transcription factors, and non-DNA binding co-regulators (co-activators and co-repressors) to target gene locus upon androgen stimulation [12]. We previously found that GATA3 facilitated androgen-liganded AR bindings and orchestrated AR-mediated transcription repression programs by recruiting co-repressors SMRT and HDAC3 across the ESCC genome. However, it remains unknown if other co-regulators associated with AR activate gene transcription in ESCC.

Metastasis is the most important prognostic factor of ESCC [13]. Accumulating evidence has shown that many kinds of factors and multiple signaling pathways were involved in ESCC cell invasion and metastasis. For example, the transcription modulator TBL1XR1 promoted ESCC metastasis via upregulation of VEGF-C [14]. LncRNA CASC9 promoted ESCC metastasis by upregulating LAMC2 expression by interacting with the CREB-binding protein [15]. Circular RNA circGSK3β promoted ESCC metastasis by augmenting β-catenin signaling [16]. Our previous studies have shown that deubiquitinating enzymes such as PSMD14, OTUB1, and USP26 promoted ESCC metastasis by stabilizing SNAIL [17,18,19]. However, the role of AR and AR signaling pathways in ESCC metastasis remains unclear.

In this study, we reported that uridine diphosphate glucuronosyltransferase family 2 member B15 (*UGT2B15*) is a novel target of AR-mediated transcriptional activation. Functional assays showed that UGT2B15 exerts a pro-metastatic function, and liganded AR promotes invasion of ESCC cells by upregulating the expression of *UGT2B15*. Mechanistically, JUN/FOSL2 directs AR-mediated *UGT2B15* transcriptional activation. We also showed that targeting the AR/FOSL2/JUN complex represses *UGT2B15* expression, thereby resulting in the suppression of ESCC cell invasion. Our study revealed the molecular mechanisms underlying the AR-driven ESCC invasion, which can be potentially targeted to inhibit ESCC metastasis. 

## 2. Materials and Methods

### 2.1. Cell Culture

This research using the human KYSE410 cell line was a gift from Dr. Yutaka Shimada (Kyoto University, Kyoto, Japan). ZEC014 cells were generously provided by Dr. Dan Su (Zhejiang University, Hangzhou, China). The human KYSE410 (RRID: CVCL_1352) and ZEC014 (provided by Dr. Dan Su) cells were maintained in RPMI 1640 supplemented with 10% fetal bovine serum and 100 units/mL streptomycin and penicillin at 37 °C in a 5% CO_2_ atmosphere. HEK293T (RRID: CVCL_0063) for lentivirus packaging was purchased from the American Type Culture Collection (ATCC) (Manassas, VA, USA) and cultured in DMEM supplemented with 10% fetal bovine serum and 100 units/mL streptomycin and penicillin according to ATCC instructions. All experiments were performed with mycoplasma-free cells.

All cell lines were authenticated by STR analysis. Firstly, DNA was extracted by a Chelex 100 reagent. Secondly, DNA was amplified with an STR multi-amplification kit (Goldeneye^TM^20A ID System, Peoplespot, Beijing, China). Then, PCR products were assayed with an ABl 3730xl DNA analyzer (Applied Biosystems, Foster City, CA, USA). Finally, data were analyzed using GeneMapper 3.2 software and then compared with the ATCC and DSMZ databases for reference matching.

### 2.2. Plasmids Construction, Lentivirus Packaging, and Infection

The full-length cDNA clone of human *UGT2B15* and control vector were purchased from GeneCopoeia (Rockville, MD, USA; EX-Z5679-Lv102 (puro) and EX-EGFP-Lv102 (puro)) and subcloned into the lentiviral vector pLVX. The shRNA oligos targeting *AR*, *FOSL2*, and *JUN*, respectively, were cloned into the pSIH-H1 vector using *EcoR* I and *BamH* I sites. To generate the lentivirus, HEK293T cells were seeded at 90% confluence and co-transfected with a lentivirus packaging system (1 μg pMD2.G and 2 μg psPAX2) and 4 μg shAR, shFOSL2, shJUN, or pLVX-UGT2B15 plasmids using Hieff Trans™ Liposomal transfection reagent (40802ES02, Yeasen Biotechnology, Shanghai, China) for 48 h. The supernatant containing lentivirus particles was collected and stored in aliquots at −80 °C. For lentivirus infection, cells were first treated with polybrene (5 µg/mL) (Sc-134220, Santa Cruz Biotechnology, Dallas, TX, USA), and then infected with the indicated lentivirus. To obtain stable cell lines, cells were selected for two weeks with puromycin (2 μg/mL) (A11138-03, Gibco, New York, NY, USA).

### 2.3. Chromatin Immunoprecipitation (ChIP) and Re-ChIP Assays and Data Analysis

KYSE410 cells were cultured in phenol red-free RPMI 1640 medium with 5% charcoal-stripped FBS for 3 days, and then treated with DHT (100 nM), R1881 (10 nM), enzalutamide (25 µM), or vehicle for 4 h, respectively. ChIP assays were performed as described previously [10]. Briefly, cells were crosslinked with 1% formaldehyde for 10 min at room temperature, and cell pellets were collected and subjected to sonication. The sheared chromatin was then diluted and pre-cleared with the indicated IgG followed by immunoprecipitation with 4 μg of specific antibodies at 4 °C overnight (anti-c-Jun, abcam, Cambridge, UK, ab31419; anti-FOSL2 CST, Boston, MA, USA, #19967S; anti-androgen receptor, Millipore, Burlington, MA, USA, PG-21, Cat. No. 06-680). Protein A-Sepharose beads were added and incubated for at least 1 h at 4 °C with rotation. The beads were then washed sequentially for 10 min each in TSE I (0.1% SDS, 1% Triton X-100, 2 mM EDTA, 20 mM Tris-HCl, pH 8.1, 150 mM NaCl), TSE II (0.1% SDS, 1% Triton X-100, 2 mM EDTA, 20 mM Tris-HCl, pH 8.1, 500 mM NaCl), and buffer III (0.25 M LiCl, 1% NP-40, 1% deoxycholate, 1 mM EDTA, 10 mM Tris-HCl, pH 8.1) and finally twice with TE buffer. Chromatin complexes were eluted with elution buffer (1% SDS, 0.1 M NaHCO_3_) and crosslinking was reversed at 65 °C overnight. DNA fragments were purified with the QIAquick PCR purification kit (Qiagen, Venlo, Netherlands, cat. no. 28104) and used for quantitative PCR reactions with SYBR Premix Ex Taq reagents. For Re-ChIP assays, the first immunoprecipitated chromatin complexes were washed, eluted with 10 mM dithiothreitol at 37 °C for 30 min, and diluted 50-fold with ChIP dilution buffer. The second immunoprecipitations were then performed. Each ChIP or Re-ChIP assay was repeated at least three times independently. Primers used for ChIP-qPCR are listed in Supplementary Table S1.

We have previously performed chromatin immunoprecipitation combined with next-generation sequencing (ChIP-seq) to identify the genome-wide AR binding sites in KYSE410 cells (accessed on 21 October 2020) [10]. Raw reads of AR ChIP-seq generated from KYSE410 cells were aligned to the human reference genome (hg19) using Bowtie 2.0 with default parameter settings [20,21]. To find enriched motifs for AR binding regions under both vehicle and R1881 treatment, we performed motif analysis using HOMER (Hypergeometric Optimization of Motif Enrichment, v4.7.2, http://homer.ucsd.edu/homer/) with default parameters using AR ChIP-Seq data. The command was ‘findMotifGenome.pl <peak file> <genome> <output directory> -size 200’.

### 2.4. Western Blotting

Western blotting was performed as previously described [10]. Cells were collected and lysed on ice using RIPA lysis buffer with 1× proteinase inhibitor cocktail (Roche, Basel, Switzerland) for 30 min. The protein concentration was determined using the Bicinchoninic acid (BCA) assay (Thermo Fisher Scientific, Shanghai, China, cat. no. 23225). The proteins were resolved on 10% SDS-PAGE and transferred onto PVDF membranes. The membranes were blocked with 5% skim milk solution at room temperature for 1 h and then incubated with the primary antibodies (anti-UGT2B15, abcam, ab89274; anti-AR, Millipore, 06-680; anti-FOSL2, CST, #19967S; anti-c-Jun, abcam, ab31419; β-actin, Sigma, St. Louis, MO, USA, A5316) at 4 °C overnight. Following incubation with secondary antibodies at room temperature for 1 h, immunoblots were visualized using an enhanced chemiluminescent substrate kit (Thermo Fisher Scientific, 32109 or 34095) and ImageQuant LAS 4000 system (GE Healthcare, Woburn, MA, USA).

### 2.5. Invasion Assays

The transwell upper chambers (3422, Costar, Corning Inc., Corning, NY, USA) were precoated with Matrigel (354248, Corning Inc.) for the invasion assay. KYSE410 or ZEC014 cells with the indicated lentivirus infection or drugs treatment were harvested and plated into the upper chamber in serum-free medium, and RPMI 1640 media containing 10% fetal bovine serum or indicated drugs were added into the bottom chamber. After 24–48 h, cells in the upper chamber were fixed with methanol and stained with 0.5% crystal violet, and cells from three random microscopic fields were counted and statistically analyzed. The experiments were biologically replicated three times. AR ligands or chemicals were purchased from the following companies: DHT (Sigma, A-8380), R1881 (Sigma, R0908), enzalutamide (Selleckchem, Houston, TX, USA, S1250), and T5224 (Selleckchem, S8966).

### 2.6. Cell Proliferation Assays

KYSE410 or ZEC014 cells (1.5 × 10^3^/well) were seeded into 96-well plates and cultured in a humidified atmosphere of 5% CO_2_ at 37 °C. The proliferation of indicated cells with lentiviral infection or drug treatment was detected using a 100 μL medium containing 10 μL CCK-8 reagents. After 1 h of incubation at 37 °C, the absorbance was measured at 450 nm. The experiments were biologically replicated three times.

### 2.7. Transfection of siRNA

The transfection was carried out using Hieff Trans™ Liposomal transfection reagent and Opti-MEM medium according to the Dharmacon’s protocol (Lafayette, CO, USA). The siRNAs used in this study were purchased from Dharmacon, including ON-TARGETplus Human UGT2B15 siRNA (L-020194-02-0005), ON-TARGETplus Human AR siRNA (L-003400-00-0020), and ON-TARGETplus Non-targeting Control Pool (D-001810-10-20).

### 2.8. RNA Extraction and RT-qPCR

Total RNA was isolated from cells using TRIzol reagent (Invitrogen, Carlsbad, CA, USA). Reverse transcription was performed using the PrimeScript Master Mix according to the manufacturer’s instructions (Takara Bio Inc., Kusatsu, Japan). RT-qPCR was carried out with an Applied Biosystems™ QuantStudio™ 5 using SYBR reagents (Thermo Fisher Scientific, A25742). Human β-actin was used as an internal control. The primer sequences used are listed in Supplementary Table S1.

### 2.9. RNA-seq and Data Analysis

Total RNA was extracted from stable *UGT2B15*-overexpressing KYSE410 cells and control cells. The cDNA library was generated using the VAHTS mRNA-seq v3 Library Prep Kit (Vazyme, Nanjing, China) according to the manufacturer’s instructions at Mingma Technologies Co., Ltd. (Shanghai, China). After PCR amplification for DNA enrichment, the AMPure XP beads (Beckman, Brea, CA, USA) were used to clean up the target fragments of 200–300 bp. After library construction, the Qubit 2.0 fluorometer dsDNA HS assay (Thermo Fisher Scientific) was used to quantify the concentration of the libraries, while the size distribution was analyzed using Agilent BioAnalyzer (Agilent, Santa Clara, CA, USA). Sequencing was performed using an Illumina Novaseq 6000 following Illumina-provided (San Diego, CA, USA) protocols for 2 × 150 paired-end sequencing at Mingma Technologies Co., Ltd. in Shanghai. For RNA-seq analysis, STAR (v2.4.2a) software allows rapid and accurate localization of RNA sequences to gene regions, and the resulting BAM files were used for downstream analysis [22]. RSEM (v1.2.29) allows precise quantification of gene or transcript expression from RNA-seq data [23]. We used the raw read counts table as the input file, applied the edgeR package to normalize the expression TMM (trimmed mean of M values) and CPM (counts per million reads mapped), and then screened genes with CPM expression values greater than 1 in at least one sample for differential expression analysis. RNA-seq data have been deposited in the GEO database under the access code GSE229393.

### 2.10. Animal Experiments

All animal studies were approved by the Institutional Animal Care and Use Committee of Cancer Hospital, Chinese Academy of Medical Sciences (NCC2020A097). KYSE410 cells stably expressing *UGT2B15* or control cells were expanded and the culture medium was replaced without puromycin two days before injection. For the experimental model for lymph node metastasis, each 6-week-old male Balb/c nude mouse was subcutaneously injected with 1 × 10^6^ cells in 20 µL PBS at foot pads. After 2 months of injection, mice were sacrificed and popliteal lymph nodes were dissected, paraffin-embedded, and H&E stained to examine the metastatic lesions. 

### 2.11. Statistical Analysis

The differences between the two groups were analyzed using the unpaired *t*-test, and the differences among three or more groups were analyzed using one-way ANOVA; *p* < 0.05 was considered statistically significant.

### 2.12. Survival Analysis

TCGA-ESCA expression data (fragments per kilobase of exon model per million mapped fragments, FPKM) and the corresponding clinical data were obtained from the UCSC Xena database (http://xena.ucsc.edu/). We selected samples of esophageal squamous carcinoma from TCGA-ESCA, including 70 males and 12 females. The clinicopathological characteristics are listed in Supplementary Table S2. After excluding a missing value, the overall survival time of 69 male ESCC patients was listed in Supplementary Table S3. Correlations between AR and UGT2B15 were identified by the Spearman correlation analysis. We built a patient-level regulatory axis activity score, incorporating AR, FOSL2, JUN, and UGT2B15, using ‘gsva’ method from the ‘GSVA’ R package. For survival analysis, we identified that the cut point of the axis activity score is 0.440469 by ‘surv_cutpoint’ method in ‘survminer’ R package. Then, we utilized the activity score to categorize male patients from the TCGA-ESCC cohort into high- and low-activity groups. A log-rank test was used to compare the differences between the subgroups.

## 3. Results

### 3.1. Expression of UGT2B15 Is Upregulated in ESCC and Androgen-Liganded AR Activates UGT2B15 Transcription

**Figure 1:**
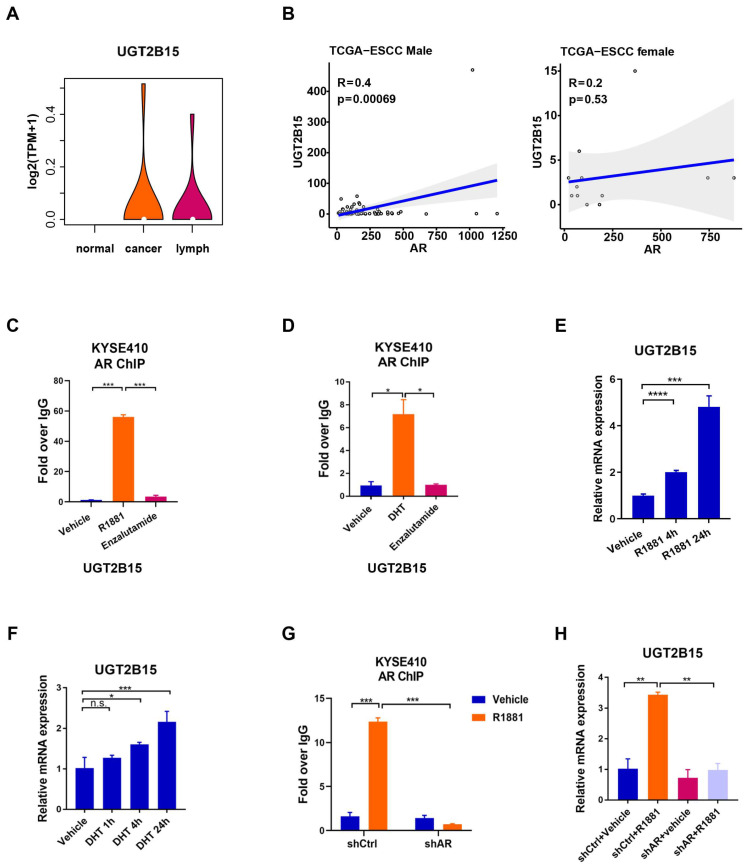
Analysis of the expression of uridine diphosphate glucuronosyltransferase family 2 member B15 (*UGT2B15*) in esophageal squamous cell carcinoma (ESCC) clinical samples and androgen-liganded androgen receptor (AR) activates *UGT2B15* transcription. (**A**) Analysis of the expression UGT2B15 in primary ESCC (*n* = 8), metastatic lymph node cancer (*n* = 14), and adjacent normal esophageal tissues (*n* = 18). (**B**) Analysis of the correlation between *UGT2B15* and *AR* expression in male (**A**) and female (**B**) ESCC patients in the TCGA database, respectively. (**C**) Standard ChIP was performed to detect the occupancy of AR within the regulatory regions of the *UGT2B15* locus in KYSE410 cells treated with vehicle, R1881 (10 nM), or enzalutamide (25 μM) for 4 h. (**D**) Standard ChIP was performed to detect the occupancy of AR within the regulatory regions of the *UGT2B15* locus in KYSE410 cells treated with vehicle, DHT (100 nM), or enzalutamide (25 μM) for 4 h. (**E**) The expression of *UGT2B15* at the mRNA level was analyzed by qRT-PCR in KYSE410 cells treated with R1881 (10 nM) at 4 h or 24 h, respectively. (**F**) The expression of *UGT2B15* at the mRNA level was analyzed by qRT-PCR in KYSE410 cells treated with DHT (100 nM) at 1 h, 4 h, or 24 h, respectively. (**G**) Standard ChIP was performed to analyze AR occupancy on the regulatory regions of the *UGT2B15* locus in AR-depleted KYSE410 cells and control cells with R1881 (10 nM) treatment for 4 h. (**H**) The expression of *UGT2B15* at the mRNA level was analyzed by qRT-PCR in AR-depleted KYSE410 cells and control cells treated with R1881 (10 nM) for 24 h. n.s., no significance, * *p* < 0.05, ** *p* < 0.01, *** *p* < 0.001, **** *p* < 0.0001.

Integrative AR ChIP-seq and RNA-seq analysis identified genes induced by R1881 treatment in ESCC cells [10]. In particular, we focused on the *UGT2B15* gene, which has been recognized to control the metabolism and homeostasis of androgen in the human prostate [24]. Since a previous study reported that AR can bind to the promoter of *UGT2B15* and *UGT2B17* [25], we examined the binding of AR at the *UGT2B15* and *UGT2B17* loci and the expression of *UGT2B15* and *UGT2B17* upon R1881 treatment based on ChIP-Seq and RNA-Seq data. The results showed that R1881 treatment induced AR binding at the *UGT2B15* locus and increased *UGT2B15* expression. In contrast, no AR binding at the *UGT2B17* locus was observed, and R1881 treatment also had no effect on *UGT2B17* expression (Supplementary Figure S1A–C). We next sought to explore the clinical relevance of UGT2B15 expression in ESCC. In a transcriptome dataset of 18 paired ESCC patients, which included 18 adjacent normal esophageal tissues, 18 primary ESCC, and 14 metastatic lymph node cancer samples [26], we observed that the *UGT2B15* was exclusively expressed in tumor tissues and metastatic lymph node samples (Figure 1A). We further used the TCGA database to analyze the correlation between *UGT2B15* and *AR* in male and female ESCC patients, respectively. Bioinformatic analyses revealed that the mRNA level of *AR* was positively correlated with *UGT2B15* in male ESCC but not in female ESCC, implying *AR* may regulate the transcription of *UGT2B15* in male ESCC (Figure 1B). We next performed AR ChIP assays to examine the occupancy of AR on the regulatory region of *UGT2B15* (Figure S1D). Treatment with DHT (dihydrotestosterone, a physiological androgen) or R1881 (a synthetic androgen) significantly increased AR binding to the regulatory region of *UGT2B15*, which was antagonized by enzalutamide (a 2nd generation AR antagonist) (Figure 1C,D). Consistently, the mRNA level of *UGT2B15* was upregulated upon either DHT or R1881 treatment for 4 h and further increased upon prolonged exposure for 24 h (Figure 1E,F). Moreover, androgen-induced AR binding and subsequent *UGT2B15* upregulation were completely abolished in AR-depleted KYSE410 cells (Figure 1G,H).

### 3.2. JUN/FOSL2 Directs Androgen-Induced UGT2B15 Transcription

**Figure 2:**
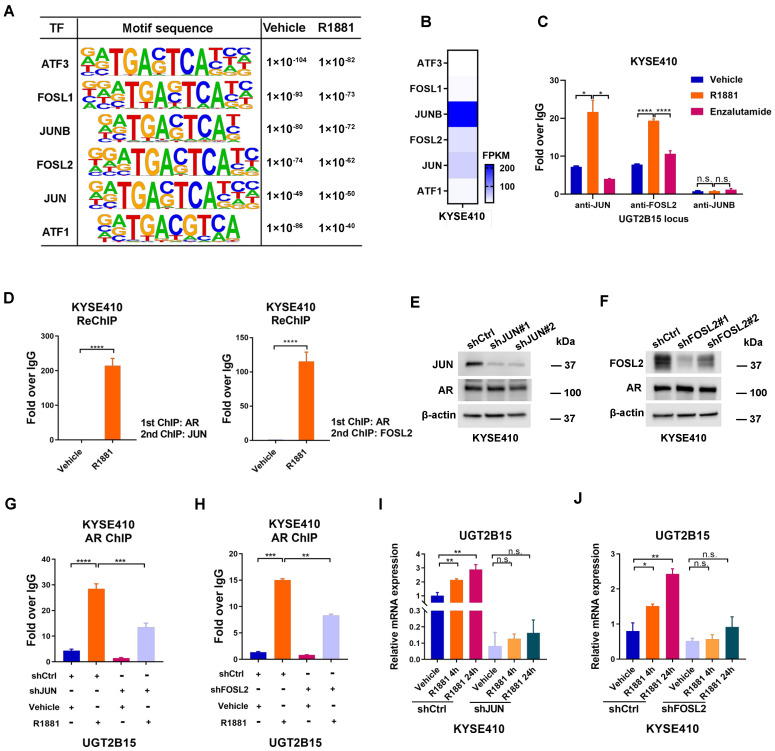
JUN/FOSL2 mediates androgen-induced *UGT2B15* transcription. (**A**) Motif analysis of AR binding sites under both vehicle and R1881 treatment was shown. The highly enriched AP-1 family members were presented. (**B**) Fragments per kilobase of exon model per million mapped fragments (FPKM) of identified AP-1 family members were shown. (**C**) KYSE410 cells were cultured for 3 days in phenol red-free RPMI 1640 medium with 5% charcoal-stripped FBS, and then treated with R1881 (10 nM) or enzalutamide (25 µM) for 4 h. Standard ChIP was performed to examine JUN, FOSL2, and JUNB occupancy on the regulatory region of the *UGT2B15* locus. (**D**) Re-ChIP assay was performed to detect the co-occupancy of AR and JUN (left) or FOSL2 (right) on the regulatory region of the *UGT2B15* locus. (**E**,**F**) Establishment of KYSE410 cells with stable knockdown of JUN (**E**) or FOSL2 (**F**). Knockdown efficiency was confirmed by Western blotting analysis. Full Western Blot images can be found in the Supplementary Materials. (**G**,**H**) JUN-depleted (**G**) or FOSL2-depleted (**H**) KYSE410 cells and control cells were treated with R1881 (10 nM) for 4 h. Standard ChIP was performed to analyze JUN or FOSL2 occupancy on the regulatory regions of *UGT2B15*. (**I**,**J**) JUN-depleted (**I**) or FOSL2-depleted (**J**) KYSE410 cells and control cells were treated with R1881 (10 nM) treatment for 4 h or 24 h. The expression of *UGT2B15* at the mRNA level was examined by qRT-PCR analysis. n.s, no significance, * *p* < 0.05, ** *p* < 0.01, *** *p* < 0.001, **** *p* < 0.0001.

Given that AR needs to recruit a number of co-regulators to modulate gene transcription [27], we next conducted motif analysis within AR direct binding regions using our previously published AR ChIP-Seq data [10]. Interestingly, we found that motifs of multiple AP-1 family members were significantly enriched in both basal and R1881-treated conditions (Figure 2A), indicating that the AP-1 transcription factor may play important and collaborative roles in AR genomic functions in ESCC cells. To determine which AP-1 subunits were involved in regulating AR-mediated *UGT2B15* transcription, we first examined the mRNA levels of AP-1 family members based on RNA-seq data from KYSE410 cells [10]. Among the six AP-1 subunits, *JUNB*, *FOSL2*, and *JUN* were the most abundant transcripts in KYSE410 cells (Figure 2B). To further confirm whether these factors are collectively bound to regulatory elements of *UGT2B15*, ChIP analysis was carried out at both vehicle and R1881 treated conditions. The occupancy of JUNB at the *UGT2B15* locus was barely detectable, while the enrichment of JUN and FOSL2 at the *UGT2B15* locus was observed at the basal levels, which was further increased upon R1881 treatment (Figure 2C). In contrast, enzalutamide treatment repressed the binding of either JUN or FOSL2. These data suggested that JUN and FOSL2 can bind to the *UGT2B15* locus in ESCC cells. 

To further confirm the roles of JUN and FOSL2 in AR transcriptional regulation, we performed a sequential ChIP assay. We found that JUN or FOSL2 formed a regulatory complex with liganded AR at the *UGT2B15* locus (Figure 2D). To determine the role of JUN or FOSL2 in AR-mediated transcriptional regulation on *UGT2B15*, we knocked down the expression of JUN and FOSL2 to perform AR ChIP and gene expression analysis. Silencing of JUN and FOSL2 significantly impaired R1881-induced AR binding to the *UGT2B15* locus without affecting AR protein levels (Figure 2E–H), resulting in the inhibition of R1881-induced *UGT2B15* gene expression (Figure 2I,J). These results suggested that JUN/FOSL2 is essential for AR binding to the regulatory region of *UGT2B15* and for the transcriptional activation of the *UGT2B15* gene in the presence of hormones.

### 3.3. UGT2B15 Promotes Metastasis in ESCC

**Figure 3:**
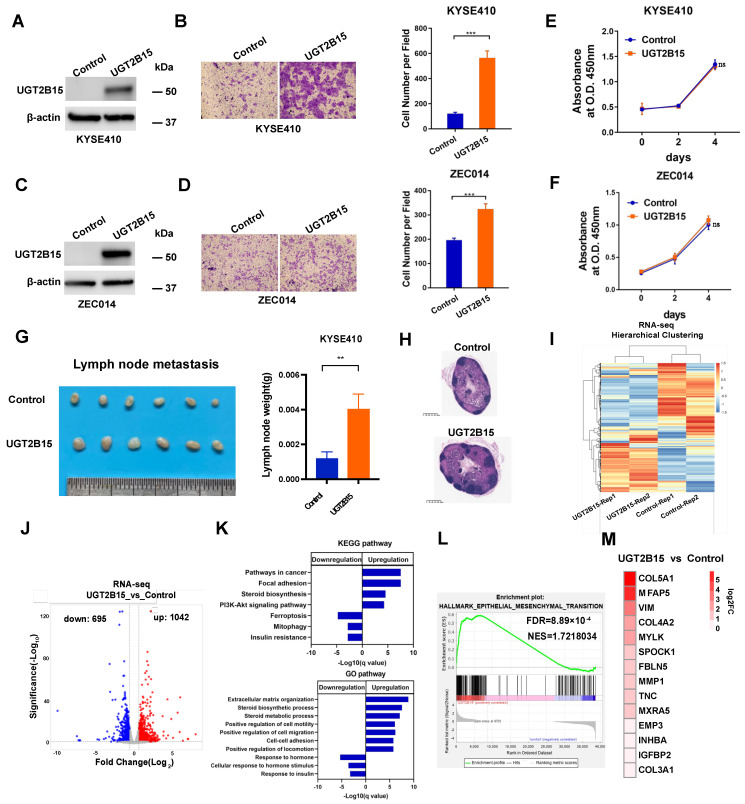
UGT2B15 promotes metastasis in ESCC. (**A**,**C**) Ectopic overexpression of UGT2B15 in KYSE410 cells (**A**) and ZEC014 cells (**C**) was examined by Western blotting. (**B**,**D**) A transwell assay was performed to evaluate the effect of UGT2B15 overexpression on the invasion of KYSE410 cells (**B**) and ZEC014 cells. Full Western Blot images can be found in the Supplementary Materials (**D**). Representative images (left panel) and statistical analysis (right panel) were shown. The experiments were biologically replicated three times. (**E**,**F**) The effect of UGT2B15 overexpression on the proliferation of KYSE410 cells (**E**) and ZEC014 cells (**F**) was assessed by CCK-8 assay. The experiments were biologically replicated three times. (**G**). The popliteal lymph nodes from the control or UGT2B15 overexpression groups were photographed (left panel) and the statistical analysis was shown (right panel) (*n* = 6). (**H**) Representative H&E staining of popliteal lymph nodes from the indicated group. Scale bars, 625 μm. (**I**,**J**) Heatmap (**I**) and volcano plot (**J**) of differentially expressed genes between KYSE410 cells stably expressing UGT2B15 and control cells. (|Log2FC| > 0.6, FDR < 0.05). (**K**) Pathway analysis of differentially expressed genes between KYSE410 cells stably expressing UGT2B15 and control cells. (**L**) Gene set enrichment analysis (GSEA) was carried out on the upregulated genes by UGT2B15 overexpression. (**M**) The expression of UGT2B15 upregulated genes was further validated by qRT-PCR analysis. ns, no significance, ** *p* < 0.01, *** *p* < 0.001.

Given that metastasis mainly accounted for poor clinical ESCC outcomes [28], we next investigated if UGT2B15 signaling played a role in the metastasis of ESCC cells. To gain more insights into the function of UGT2B15 in regulating ESCC cell invasion, we established KYSE410 and ZEC014 cells stably overexpressing *UGT2B15* and assessed how UGT2B15 influenced the cellular phenotypes in vitro and in vivo. We found that ectopic overexpression of *UGT2B15* greatly enhanced the invasiveness of KYSE410 and ZEC014 cells (Figure 3A–D). Interestingly, in contrast with the role of UGT2B15 in cell proliferation in hormone-sensitive prostate cancer cells [29], no significant effects on cell proliferation were observed between control and *UGT2B15*-overexpressing KYSE410 and ZEC014 cells (Figure 3E,F). To investigate whether the effect of UGT2B15 on cell invasion is dependent on AR, we transfected siRNAs targeting AR into UGT2B15-overexpressing KYSE410 cells and then performed a cell invasion assay. AR knockdown did not affect the promotion of cell invasion of UGT2B15 overexpression (Figure S1E). To further characterize if UGT2B15 can promote metastasis in vivo, we subcutaneously inoculated control and UGT2B15-overexpressing KYSE410 cells into the foot pads of male Balb/c nude mice (N = 6 in each group). After 2 months, popliteal lymph nodes (LNs) were collected and weighed. Consistent with the findings that *UGT2B15* strongly induced invasive phenotype in vitro, the popliteal LNs in the *UGT2B15* overexpression group were larger and heavier than those in the control group, demonstrating the functional role of UGT2B15 in driving LN metastasis from primary tumors at the inoculation site (Figure 3G). H&E staining showed that the number of lymph node metastases in the *UGT2B15* overexpression group was more than that in the control group (Figure 3H). To delineate the molecular mechanism by which UGT2B15 promoted metastasis of ESCC cells, we performed RNA-seq to identify the differentially expressed genes (DEGs) between *UGT2B15* overexpressed and control KYSE410 cells. Unsupervised hierarchical clustering of RNA-seq data clearly revealed the distinct gene expression patterns in control cells and *UGT2B15*-overexpressing KYSE410 cells (Figure 3I). *UGT2B15* overexpression significantly upregulated 1042 genes and downregulated 695 genes (Figure 3J). Kyoto Encyclopedia of Genes and Genomes (KEGG) and Gene Ontology (GO) pathways analyses showed that both UGT2B15-induced or repressed genes were commonly associated with steroid hormone response (Figure 3K), which was consistent with the function of UGT2B15 in regulating the homoeostasis of steroid hormones in prostate cancer cells [24]. As expected, upregulated genes were preferentially enriched in cancer-relevant biological processes, including cell motility (Figure 3K). Notably, gene set enrichment analysis (GSEA) revealed the significant enrichment of canonical gene signatures ‘epithelial–mesenchymal transition’ implicated in cancer metastasis (Figure 3L). The expression of genes enriched in cancer metastasis was also validated by qRT-PCR analysis (Figure 3M). Overall, these data demonstrated that UGT2B15 promotes metastasis by upregulating pro-metastasis genes in ESCC cells.

### 3.4. Androgen-Liganded AR and AP-1 Promote ESCC Cell Invasion

**Figure 4:**
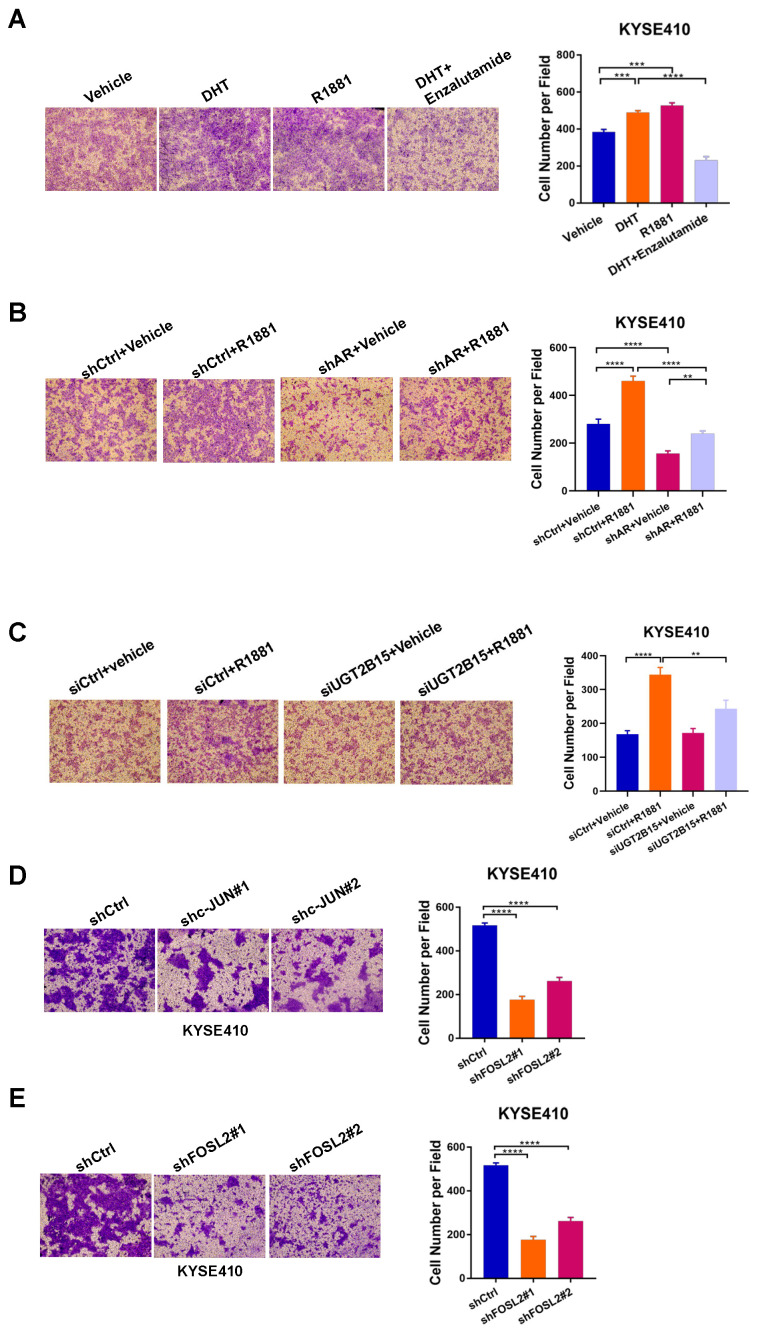
AR promotes ESCC cell invasion by regulating UGT2B15. (**A**) KYSE410 cells were cultured in phenol-red free RPMI 1640 medium supplemented with 5% charcoal-stripped FBS for three days, followed by treatment of DHT (100 nM), R1881 (10 nM), or both DHT (100 nM) and enzalutamide (25 μM) for 24 h. Transwell assays were then performed. Representative images (left panel) and statistical analyses (right panel) were shown. (**B**) Transwell assay was performed to evaluate the effect of R1881 (10 nM) treatment on the invasion ability of KYSE410 cells expressing shRNA targeting AR (shAR) and control cells (shCtrl). Representative images (left panel) and statistical analyses (right panel) were shown. (**C**) Transwell assay was performed to evaluate the effect of R1881 (10 nM) treatment on the invasion ability of KYSE410 cells transfected with siRNA targeting *UGT2B15* or control siRNA for 48 h. Representative images (left panel) and statistical analyses (right panel) were shown. (**D**,**E**) Representative images and statistical analyses of transwell assays in JUN-depleted (**D**) or FOSL2-depleted (**E**) and control KYSE410 cells. The above experiments were biologically replicated three times. ** *p* < 0.01, *** *p* < 0.001, **** *p* < 0.0001.

We previously reported that higher expression of AR protein was correlated with shorter overall survival and disease-free survival of ESCC patients [10]. We next interrogated if AR signaling played a role in the invasion of ESCC cells. Treatment of DHT or R1881 significantly promoted the invasive ability of KYSE410 and ZEC014 cells, of which AR is highly expressed. Conversely, enzalutamide significantly diminished the effect of DHT and inhibited invasion in both cells (Figure 4A and Figure S1F–H). Consistently, genetic knockdown of AR impaired androgen-enhanced cell invasion compared to control cells, suggesting that AR is required for hormone-induced invasion in ESCC (Figure 4B and Figure S1I). To investigate whether UGT2B15 contributes to the AR-promoted invasive phenotype, we silenced *UGT2B15* expression in KYSE410 cells and then performed a cell invasion assay with R1881 or vehicle treatment. Silencing of *UGT1B15* significantly impaired R1881-induced cell invasiveness in KYSE410 cells, suggesting that UGT2B15 may be a critical downstream effector responsible for AR-driven cell invasion in ESCC (Figure 4C and Figure S1J).

Consistent with the role of JUN/FOSL2 directing AR transcriptional activation of *UGT2B15* in ESCC, we found that depletion of JUN and FOSL2 significantly suppressed the invasion of both KYSE410 and ZEC014 cells without affecting cell proliferation (Figure 4D,E and Figure S2A–F).

### 3.5. AP-1 Inhibitor Impairs Androgen-Induced Transcription of UGT2B15 and Invasiveness of ESCC Cells

**Figure 5:**
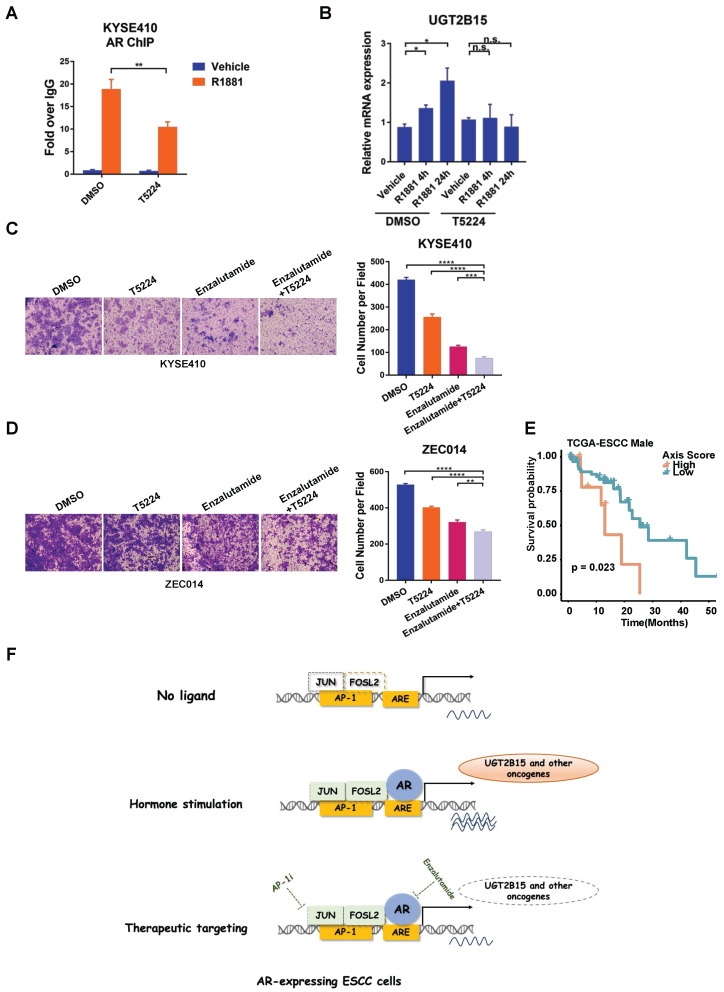
Combined inhibition of AR and AP-1 suppresses the invasiveness of ESCC cells. (**A**) KYSE410 cells were cultured in phenol red-free RPMI 1640 medium with 5% charcoal-stripped FBS for 3 days and treated with AP-1 inhibitor (T5224, 10 μM) for 24 h, followed by R1881 (10 nM) treatment for 4 h. Standard ChIP was performed to detect the AR occupancy on the regulatory regions of the *UGT2B15* gene locus. (**B**) The expression of *UGT2B15* at the mRNA level was examined by qRT-PCR analysis in KYSE410 cells treated with vehicle or 10 nM R1881 at 4 h or 24 h alone or in combination with 10 μM T5224 treatment for 4 h or 24 h. (**C**,**D**) Representative images and statistical analysis of transwell assays in KYSE410 cells (**C**) and ZEC014 cells (**D**) treated with DMSO, 10 μM T5224, 25 μM enzalutamide or a combination of 25 μM enzalutamide and 10 μM T5224 for 24 h. The experiments were biologically replicated three times. (**E**) Analysis of male ESCC patients’ survival in strata defined according to high or low activities of AR/AP-1/UGT2B15 axis (log-rank *p* = 0.023). (**F**) Proposed model of AP-1 directing AR transcriptional activation program in ESCC and potential therapeutic interventions. n.s., no significance, * *p* < 0.05, ** *p* < 0.01, *** *p* < 0.001, **** *p* < 0.0001.

Given that AP-1 can be targeted by the small molecule inhibitor T5224, we next examined whether T5224 treatment impairs androgen-induced AR binding to the regulatory region of *UGT2B15* and androgen-induced *UGT2B15* transcription. Treatment with T5224 significantly attenuated the recruitment of AR at the *UGT2B15* locus, leading to the suppression of R1881-induced *UGT2B15* expression (Figure 5A,B). These results motivated us to further investigate whether T5224 could affect enzalutamide-inhibited cell invasiveness. Co-treatment with T5224 and enzalutamide led to stronger cell invasiveness inhibition compared with T5224 or enzalutamide alone (Figure 5C). Similar results were obtained in ZEC014 cells (Figure 5D). These findings strongly suggested that simultaneously targeting AP-1 and AR can more effectively inhibit the invasiveness of AR-positive ESCC cells. Lastly, to determine the association between the AR/AP-1/UGT2B15 axis and ESCC patient survival, we built a patient-level regulatory axis activity score incorporating AR, FOSL2, JUN, and UGT2B15 expression levels, which were utilized to categorize male patients from the TCGA-ESCC cohort into high- and low-activity groups. The low-activity group exhibited a favorable survival outcome, whereas the high-activity group demonstrated a poorer survival (Figure 5E).

## 4. Discussion

Our study suggested that the androgen-liganded AR promotes invasive phenotypes of AR-expressing ESCC cells by upregulating *UGT2B15* expression. UGT2B15 is one of two UDP-glucuronosyltransferase (UGT) enzymes, which mediates androgen glucuronidation reaction to inactivate androgen signaling, thereby maintaining androgen homeostasis in the human prostate [24,30]. It has been recognized that UGT2B15 is only expressed in the AR-positive cell lines and *UGT2B15* expression is markedly repressed by AR to sustain pro-proliferative functions of androgen in prostate cancer [29,30,31]. Clinically, UGT2B15 levels are significantly reduced in hormone-naive and castration-resistant prostate cancer and hardly detected in lymph node metastases [32]. Interestingly, opposing the tumor suppressive function of UGT2B15 in prostate cancer, our data showed that the expression of *UGT2B15* is significantly upregulated in ESCC tissues compared with their matched normal esophageal epithelial tissues. Knockdown of *UGT2B15* diminished androgen-induced invasiveness, indicating that the liganded AR promotes cell invasion at least in part by activating *UGT2B15* transcription. We further demonstrated that UGT2B15 drives lymph node metastasis of ESCC by the induction of genes involved in cancer metastasis. Among the genes regulated by UGT2B15 in Figure 3M, COL5A1 (collagen type V alpha 1 chain) and MFAP5 (microfibrillar-associated protein 5) were most significantly upregulated and may be potential downstream targets of UGT2B15. Although no studies of COL5A1 and MFAP5 in ESCC have been reported, these two genes are involved in tumor metastasis in other types of tumors. Previous studies have also found that COL5A1 is a marker of EMT in tumor cells and can directly promote EMT [33]. MFAP5 is a component of extracellular elastic microfibril. Stromal MFAP5 acting as a metastasis-promoting gene and targeting stromal MFAP5 significantly decreased ovarian tumor growth and metastasis in vivo [34]. In addition, MFAP5 has been reported to promote basal-like breast cancer progression by activating the EMT program [35]. Although UGT2B15 may influence the expression of COL5A1 and MFAP5, the functional role of these two genes in UGT2B15-induced esophageal cancer cell invasion and metastasis needs to be further clarified by rescue experimental validation.

The AP-1 transcription factor is a dimeric complex consisting of basic leucine-zipper (bZIP) proteins that include JUN, FOS, ATF (activating transcription factor), and MAF (musculoaponeurotic fibrosarcoma) protein families [36]. Extracellular signals trigger the formation of AP-1 heterodimers or homodimers, which recognize specific DNA motifs to regulate the expression of genes in cell proliferation, cell transformation, differentiation, apoptosis, angiogenesis, and invasion [37]. In this study, we showed that the binding of JUN/FOSL2 is significantly enriched at the *UGT2B15* locus and further increased by R1881 treatment. JUN/FOSL2 interacted with AR on chromatin and facilitated the R1881-induced AR binding to the *UGT2B15* region. Accordingly, depletion of JUN/FOSL2 repressed R1881-induced *UGT2B15* expression. Consistent with findings that AP-1 promoted invasiveness of triple-negative breast cancer [38] and squamous cell carcinoma [39], knockdown of JUN/FOSL2 inhibited invasion of AR-expressing ESCC cells without influencing cell growth ability, suggesting that the JUN/FOSL2/AR transcriptional axis may especially exert its function in ESCC metastasis. 

T5224, a selective inhibitor of AP-1, was first developed to treat rheumatoid arthritis by blocking c-Fos/AP-1-mediated inflammatory and matrix metalloproteinase (MMP) action and has successfully advanced into phase II clinical trials [40]. Given that the AP-1 complex is extensively implicated in almost all physiological and pathological processes [41], inhibition of AP-1 by T5224 has been demonstrated to exhibit broad therapeutic effects in various disease models, including lipopolysaccharide-induced liver injury [42], endotoxin-induced acute kidney injury [43], as well as intervertebral disc degeneration [44]. Notably, T5224 has been used to prevent metastasis of oral cancer [45] and squamous cell carcinoma [46]. In the present study, we showed that the combination of AR inhibitor with T5224 exhibits a stronger inhibitory effect on cell invasion, possibly resulting from T5224-mediated inhibition of canonical pro-metastatic genes [40,45]. To sum up, our data suggested that enzalutamide-based anti-AR therapy combined with AP-1 inhibitor may have promising potential for preventing metastasis in AR-positive ESCC patients. In the future, the effect of combined inhibition of AR and AP-1 on ESCC metastasis needs to be further verified in mouse models. 

In summary, our findings suggest that androgen-induced UGT2B15 has a metastasis-promoting function in ESCC. We reveal that the molecular mechanism underlying AR upregulates transcription of the oncogenic gene UGT2B15 in ESCC and identify the AR/AP-1/UGT2B15 transcription axis as a potential target for male ESCC invasion and metastasis. Pharmacological inhibition of AR and AP-1 can effectively block the expression of UGT2B15, thus inhibiting the invasion and metastasis of male esophageal cancer (Figure 5F).

## 5. Conclusions

In this study, we revealed that AP-1 guides AR binding and collaborates with AR to upregulate target gene *UGT2B15* expression to enhance esophageal cancer cell invasion and metastasis. We further investigated the mechanism of UGT2B15 as a metastasis promoter in ESCC. This study may provide a foundation for identifying the AR/AP1/UGT2B15 transcription axis as a promising target for androgen-driven metastasis in male ESCC patients. Our findings suggest that the combination of AP-1 and AR inhibitors has therapeutic potential to prevent male ESCC metastasis.

## Data Availability

RNA-seq data have been deposited in the GEO database GSE229393 at https://www.ncbi.nlm.nih.gov/geo/query/acc.cgi?acc=GSE229393 (accessed on 5 September 2023). The GEO database is an open access resource and bona fide researchers can apply to use the GEO dataset by registering and applying at https://www.ncbi.nlm.nih.gov/geo/.

## Supplementary Materials

The following supporting information can be downloaded at: https://www.mdpi.com/article/10.3390/cancers15245719/s1, Figure S1: Androgen-liganded AR activates UGT2B15 transcription and promotes ESCC cell invasion, Figure S2: Combined inhibition of AR and AP-1 suppresses invasiveness of ESCC cells, Table S1: Primers, shRNAs used in this study, Table S2: Clinicopathological characteristics of ESCC from TCGA cohort, Table S3: Overall survival (OS) of male ESCC patients from TCGA cohort.

## Abbreviations

AR	Androgen receptor
ESCC	Esophageal squamous cell carcinoma
ChIP	chromatin immunoprecipitation
TCGA	The Cancer Genome Atlas
AP-1	Activator protein 1
EMT	Epithelial–mesenchymal transition
UGT2B15	Uridine Diphosphate Glucuronosyltransferase Family 2 Member B15
UGT2B17	Uridine Diphosphate Glucuronosyltransferase Family 2 Member B17
FOSL2	FOS-like antigen 2
JUN	Jun proto-oncogene
HDAC3	Histone Deacetylase 3
COL5A1	Collagen type V alpha 1 chain
MFAP5	Microfibrillar-associated protein 5
ATF	Activating transcription factor
MAF	musculoaponeurotic fibrosarcoma
