# Supplementary material for: Androgen Receptor/AP-1 Activates UGT2B15 Transcription to Promote Esophageal Squamous Cell Carcinoma Invasion
Source: Cancers (Basel). 2023 Dec 6;15(24):5719. doi: 10.3390/cancers15245719 (PMC10741602; doi:10.3390/cancers15245719)
Supplement: Supplementary file 1 [file cancers-15-05719-s001.zip › cancers-2638997-supplementary.pdf]

cancers

MDPI

Supplementary Material: Androgen Receptor/AP-1 Activates *UGT2B15* Transcription to Promote Esophageal Squamous Cell Carcinoma Invasion

Jiahui Cai, Furong Huang, Wenyan Gao, Tongyang Gong, Hongyan Chen and Zhihua Liu

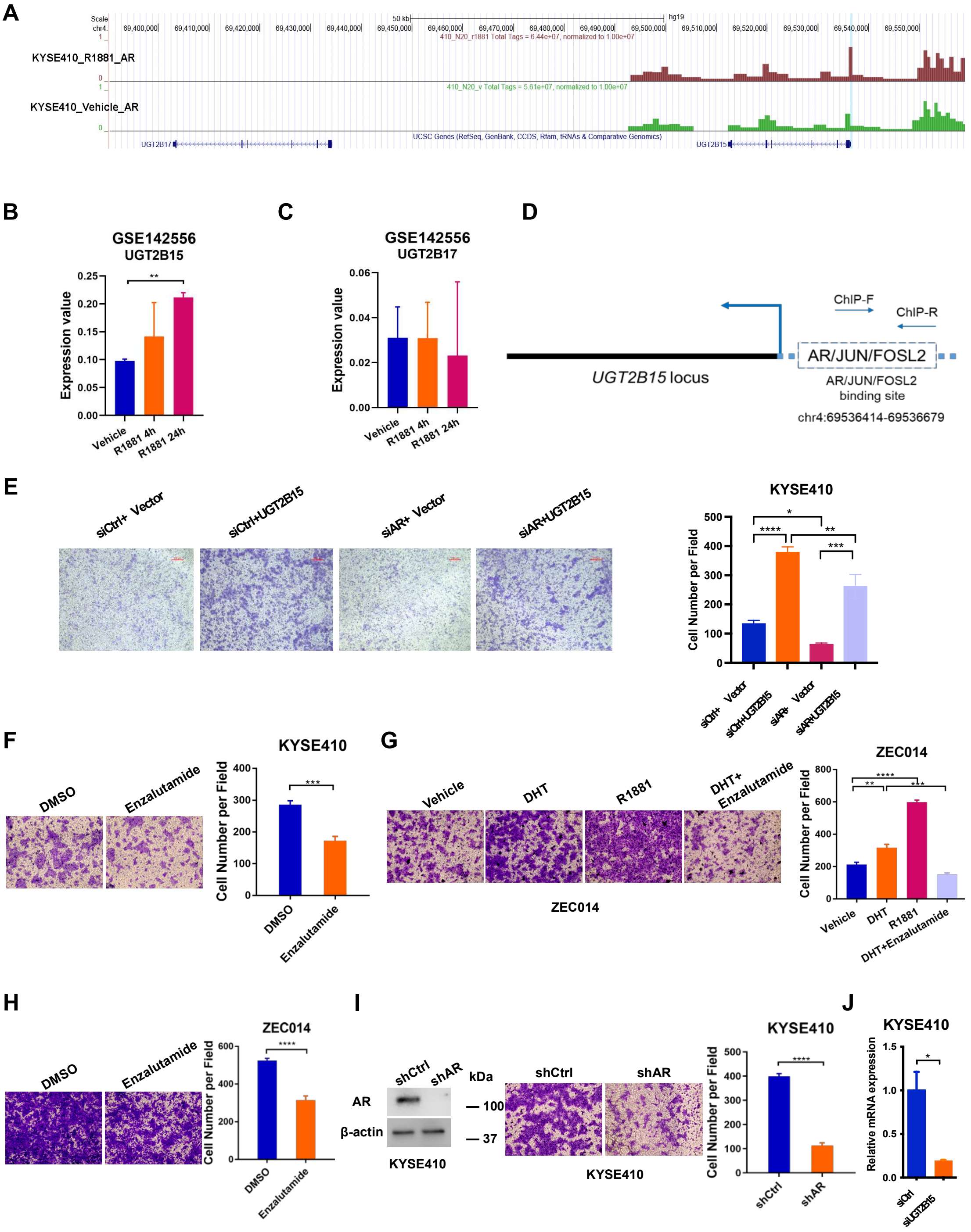

Figure S1. Androgen-liganded AR activates UGT2B15 transcription and promotes ESCC cell invasion. (**A**) Motif analysis of AR binding sites under both vehicle and R1881 treatment. (**B,C)** The expression of *UGT2B15* (**B**) and *UGT2B17* (**C**) at the mRNA level was analyzed by qRT-PCR in KYSE410 cells treated with R1881 (10 nM) at 4 h or 24 h, respectively. (**D)** schematic of gene regulatory elements, position of primers relative to binding sites of AR/JUN/FOSL2 in the promoter region of UGT2B15. (**E**) Transwell assay was performed to evaluate the independent impact of UGT2B15 on cell invasion ability. AR was depleted by siRNAs transfection in UGT2B15-overexpressing KYSE410 cells and control cells and then cell invasion assay was performed. Representative images of transwell assays (left panel) and statistical analysis (right panel) were shown. (**F,G)** Representative images and statistical analysis of transwell assay in KYSE410 cells (**E**) and ZEC014 cells (**F**) treated with DMSO or 25 μM enzalutamide for 24 h. (**H**) ZEC014 cells were cultured in phenol-red free RPMI 1640 medium supplemented with 10% Charcoal-stripped FBS for three days, followed by treatment of DHT (100 nM), R1881 (10 nM) or both DHT (100 nM) and enzalutamide (25 μM) for 24 h. Representative images (left panel) and statistical analysis (right panel) were shown. **I** AR knockdown efficiency in KYSE410 cells was confirmed by Western Blotting analysis. Transwell assay was performed to evaluate invasive ability in AR-silenced KYSE410 (shAR) and control cells (shCtrl). Representative images (left panel) and statistical analysis (right panel) were shown. (**J**) Knockdown efficiency of UGT2B15 in KYSE410 cells was confirmed by RT-qPCR. ** $p < 0.01$, *** $p < 0.001$, **** $p < 0.0001$.

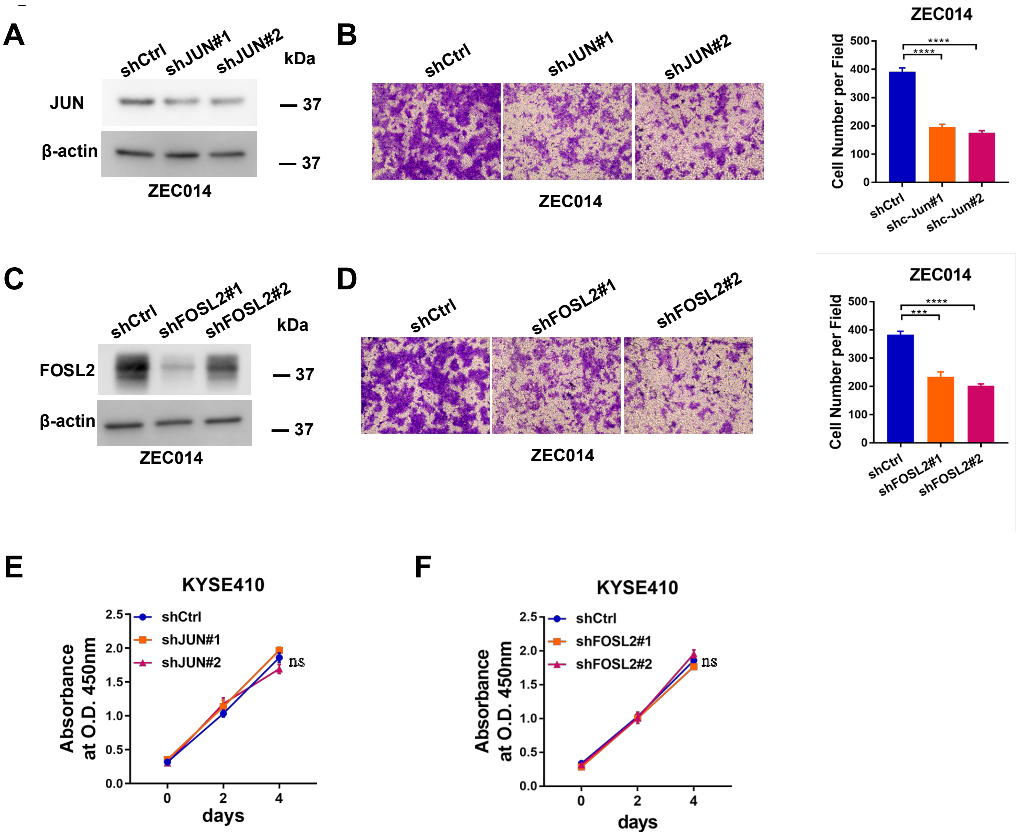

Figure S2. Combined inhibition of AR and AP-1 suppresses invasiveness of ESCC cells. (**A**) Establishment of ZEC014 cells with stable JUN depletion. Knockdown efficiency was confirmed by Western Blotting analysis. (**B**) Representative images and statistical analysis of transwell assays in *JUN*-depleted and control ZEC014 cells. (**C**) Establishment of ZEC014 cells with stable depletion of FOSL2. Knockdown efficiency was confirmed by Western Blotting analysis. (**D**) Representative Images and statistical analysis of transwell assays in *FOSL2*-depleted and control ZEC014 cells. (**E,F**) The effect of depletion of JUN (**E**) or FOSL2 (**F**) on the proliferation was assessed by CCK-8 assay in KYSE410 cells. n.s., no significance, ** $p < 0.01$, *** $p < 0.001$, **** $p < 0.0001$.

Table S1. Primers, shRNAs used in this study.

Name	Forward Primer Sequence (5′ to 3′)	Reverse Primer Sequence (5′ to 3′)
COL5A1	CTTGGCCCAAAGAAAACCCG	GTAGGTGACGTTCTGGTGGG
MFAP5	GCATCGGCCGGTTAAACAAT	TCACAGGGAGGAAGTCGGAA
VIM	GGACCAGCTAACCAACGACA	AAGGTCAAGACGTGCCAGAG
COL4A2	ATAGGAGGGCCCAAGGGATT	CAGGGTCCCCTCTATCACCA
MYLK	GCATCAAGTACATGCGGCAG	TCTCATCGAATGCCTCGTCG
SPOCK1	CTCGGTGTAATGAGGAGGGC	CTGCCAAAATCCCCTGAGGT
FBLN5	AGACGCCCCAAGATTGTTGT	CACTCGTCCACATCCACACA
MMP1	ATGTGGAGTGCCTGATGTGG	TTGTCCCGATGATCTCCCCT
TNC	GGGTCCAGATACCACCTCCT	ATCCATCCACCCCCATCAGA
MXRA5	GGAGACCCCAGATACCCACT	ACACAGTGTCTGTCTCAGCG
EMP3	CCTCTTGCTGGTGGTCTCAG	CTGACATTACTGCAGGCCCA
INHBA	TCATCACGTTTGCCGAGTCA	TGTTGGCCTTGGGGACTTTT
IGFBP2	GGCGAGGGCACTTGTGAGAA	CAGTGACCTTCTCCCGGAAC
COL3A1	GGTCTCAGTGGAGAACGTGG	TCTGTCCACCAGTGTTTCCG
UGT2B15	GGCCCACAGAATACAGCCAT	TGCATCTTTACAGAGCTTGTTACT
Actin	AGGCACCAGGGCGTGAT	GCCCACATAGGAATCCTTCTGAC
ChIP_UGT2B15	TGCATGCCAAGGAGACCAAC	GCCTCTCACTTGCCACTGTT
shCtrl	CCGG AATTCTCCGAACGTGTCACGT CTCGAG ACGTGACACGTTCGGAGAATT TTTTTG	AATTCAAAAA AATTCTCCGAACGTGTCACGT CTCGAG ACGTGACACGTTCGGAGAATT
shFOSL2#1	CCGG GCAGTGAGTATTGGAAGACTT CTCGAG AAGTCTTCCAATACTCACTGC TTTTTG	AATTCAAAAA GCAGTGAGTATTGGAAGACTT CTCGAG AAGTCTTCCAATACTCACTGC
shFOSL2#2	CCGG CACGGCCCAGTGTGCAAGATT CTCGAG AATCTTGCACACTGGGCCGTG TTTTTG	AATTCAAAAA CACGGCCCAGTGTGCAAGATT CTCGAG AATCTTGCACACTGGGCCGTG
shJUN#1	CCGG CGGACCTTATGGCTACAGTAA CTCGAG TTACTGTAGCCATAAGGTCCG TTTTTG	AATTCAAAAA CGGACCTTATGGCTACAGTAA CTCGAG TTACTGTAGCCATAAGGTCCG
shJUN#2	CCGG CCAGCGTATCTATATGGAATT CTCGAG AATTCCATATAGATACGCTGG TTTTTG	AATTCAAAAA CCAGCGTATCTATATGGAATT CTCGAG AATTCCATATAGATACGCTGG
shAR	CCGG CGAGAGAGCTGCATCAGTT CTCGAG AACTGATGCAGCTCTCTCG TTTTTG	AATTCAAAAA CGAGAGAGCTGCATCAGTT CTCGAG AACTGATGCAGCTCTCTCG

Table S2. Clinicopathological characteristics of ESCC from TCGA cohort.

Characteristics	Male (*n* = 70)	Female (*n* = 12)
OS state		
Alive	45	11
Dead	25	1
T stage		
T1	5	3
T2	23	4
T3	36	5
T4	3	0
Unknown	3	0

N stage		
N0	36	10
N1	25	1
N2	4	1
N3	1	0
Unknown	4	0
M stage		
M0	59	11
M1	3	0
Unknown	8	1
Cancer stage		
Stage I	4	3
Stage II	39	8
Stage III	21	1
Stage IV	3	0
Unknown	3	0
Radiation therapy		
Yes	24	2
No	27	8
Unknown	19	2

OS: Overall survival; T: Tumor; N: Node; M: Metastasis.

Table S3. Overall survival (OS) time of male ESCC patients from TCGA cohort.

**Sample (n = 70)**	**Overall survival (days)**
TCGA-LN-A9FO-01A	4
TCGA-IG-A50L-01A	16
TCGA-IG-A5B8-01A	24
TCGA-Z6-A9VB-01A	40
TCGA-VR-A8ET-01A	47
TCGA-Z6-A8JE-01A	64
TCGA-IG-A3YB-01A	80
TCGA-Z6-AAPN-01A	81
TCGA-Z6-A8JD-01A	104
TCGA-V5-A7RC-01B	104
TCGA-V5-A7RC-06A	104
TCGA-JY-A6FE-01A	112
TCGA-LN-A5U5-01A	136
TCGA-IG-A8O2-01A	142
TCGA-KH-A6WC-01A	191
TCGA-VR-A8EW-01A	247
TCGA-IG-A6QS-01A	303
TCGA-LN-A7HV-01A	320
TCGA-L7-A56G-01A	330
TCGA-LN-A4A9-01A	351
TCGA-LN-A7HW-01A	365
TCGA-LN-A7HY-01A	366
TCGA-IG-A97I-01A	370
TCGA-LN-A7HX-01A	372
TCGA-LN-A9FR-01A	373
TCGA-LN-A4MQ-01A	375
TCGA-LN-A8HZ-01A	375
TCGA-LN-A49P-01A	375
TCGA-LN-A5U6-01A	375
TCGA-VR-A8ER-01A	378
TCGA-LN-A49Y-01A	379
TCGA-LN-A4A1-01A	383

TCGA-LN-A4A4-01A	383
TCGA-LN-A49X-01A	384
TCGA-LN-A49M-01A	385
TCGA-LN-A4A3-01A	388
TCGA-IG-A625-01A	390
TCGA-LN-A9FQ-01A	391
TCGA-LN-A49S-01A	400
TCGA-LN-A7HZ-01A	401
TCGA-LN-A49W-01A	403
TCGA-LN-A8I0-01A	407
TCGA-LN-A49O-01A	408
TCGA-XP-A8T8-01A	437
TCGA-IG-A97H-01A	441
TCGA-LN-A49U-01A	467
TCGA-V5-AASV-01A	467
TCGA-L5-A88S-01A	471
TCGA-LN-A4A8-01A	472
TCGA-VR-AA7I-01A	484
TCGA-IG-A51D-01A	518
TCGA-VR-A8EZ-01A	553
TCGA-VR-A8EU-01A	557
TCGA-IG-A4P3-01A	567
TCGA-IG-A3YC-01A	612
TCGA-S8-A6BW-01A	620
TCGA-IG-A3YA-01A	632
TCGA-L5-A8NQ-01A	650
TCGA-LN-A4A5-01A	681
TCGA-XP-A8T6-01A	763
TCGA-L5-A88W-01A	764
TCGA-LN-A5U7-01A	768
TCGA-VR-A8EO-01A	785
TCGA-VR-A8EP-01A	824
TCGA-VR-A8EX-01A	855
TCGA-IG-A3QL-01A	1071
TCGA-JY-A6FG-01A	1263
TCGA-JY-A6FA-01A	1361
TCGA-VR-A8Q7-01A	1590
TCGA-L5-A43J-01A	-

Overall survival: the length of time from the initial diagnosis of cancer until death/the last follow-up.

410 shc-JUN and 30 shAR-AT 2

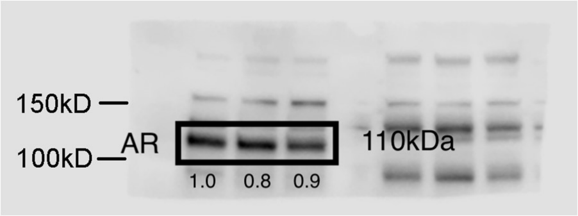

410 shc-Jun-actin

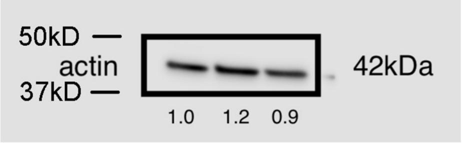

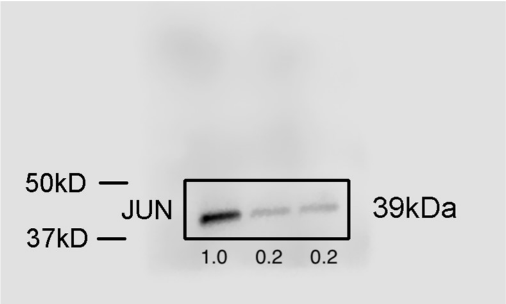

410 shc-JUN-c-jun 1-1

Figure S3. Full Western Blot images for Figure 2E. Densitometry readings for each band: 410 shc-JUN and 30 shAR-AR 2: 47146.19; 47852.97; 40228.24; 410 shc-Jun-actin: 46321.33; 55671.99; 42323.87; 410 shc-JUN-c-Jun 1-1: 49532.81; 14380.71; 11260.95.

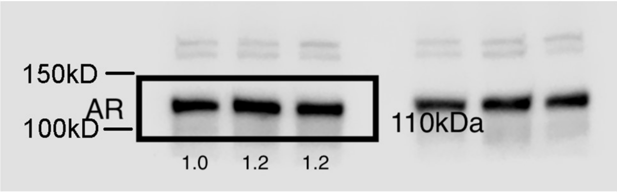

3 410 shFra2 to detect AR-2

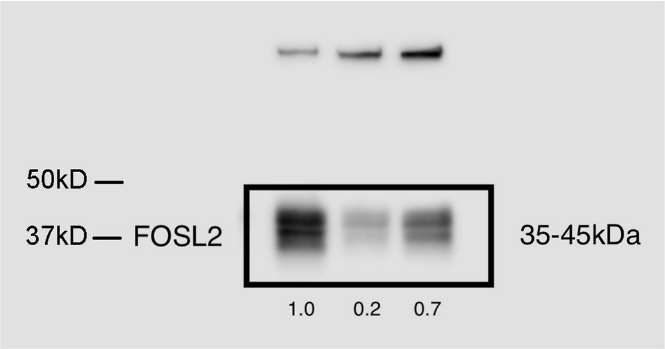

410shFra2-1

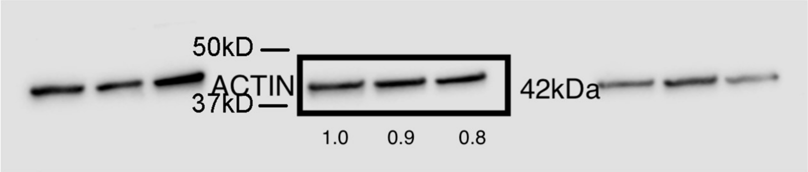

Actin 1 2 3-2

Figure S4. Full Western Blot images for Figure 2F. Densitometry readings for each band: 3 410 shFra2 to detect AR-2: 46900.48; 53584.75; 46995.36; 410shFra2-1: 49421.92; 11522.05; 27190.60; Actin 1 2 3-2: 56049.50; 52845.87; 47014.82.

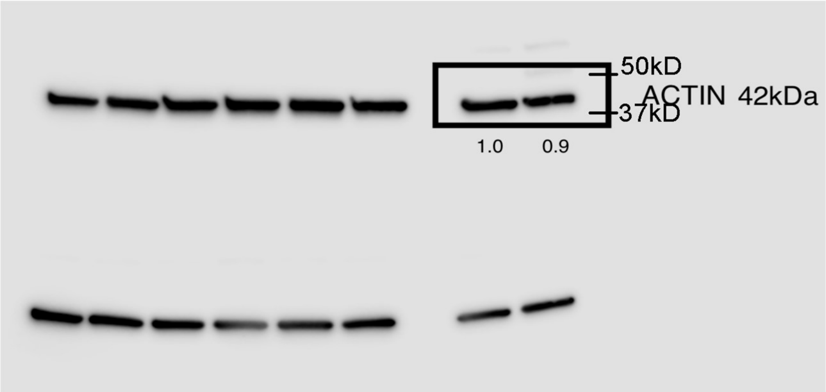

1 2 actin 5

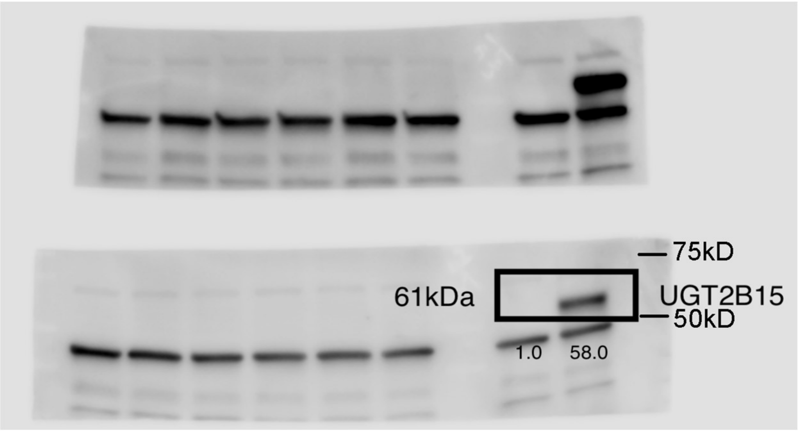

1 2 OE UGT 2

Figure S5. Full Western Blot images for Figure 3A. Densitometry readings for each band: 1 2 actin 5: 85136.92; 78098.56; 1 2 OE UGT 2: 1471.68; 78251.76.

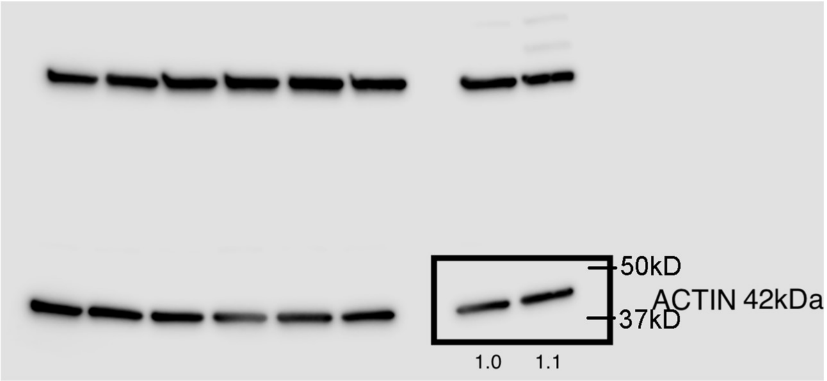

1 2 actin 5

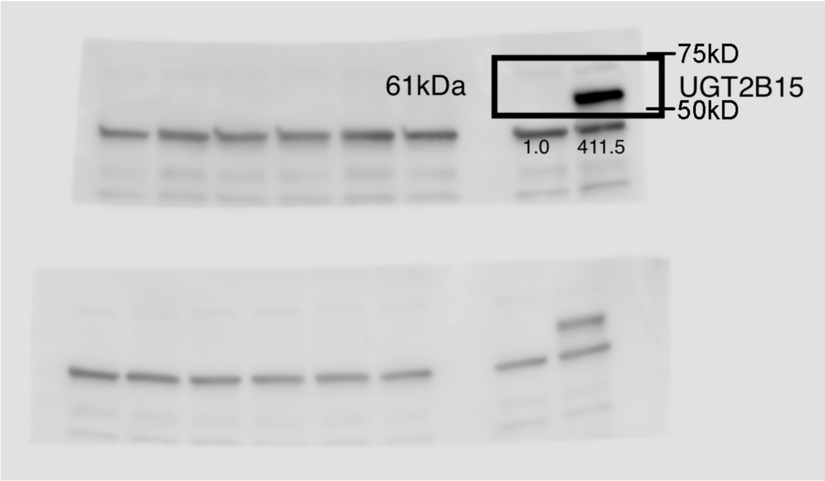

1 2 OE UGT 1

Figure S6. Full Western Blot images for Figure 3D. Densitometry readings for each band: 1 2 OE UGT 1: 181.54; 81535.69; 1 2 actin 5: 70505.44; 76948.59.

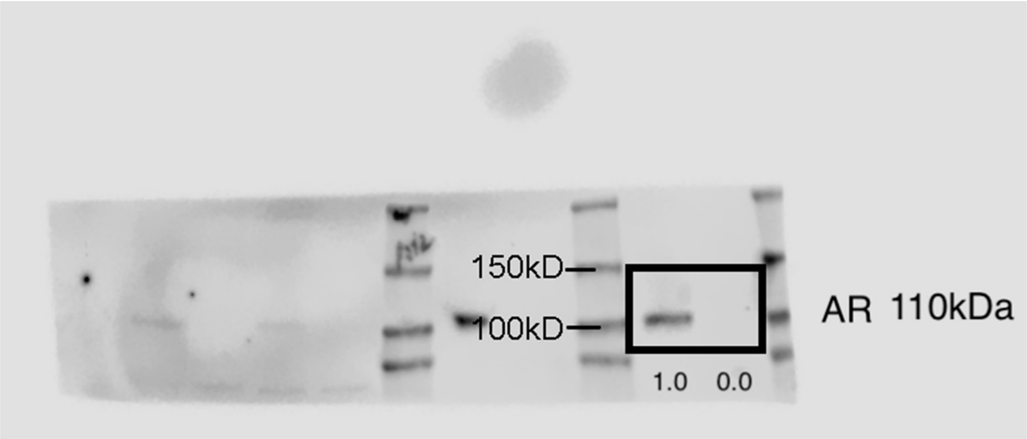

410 shAR

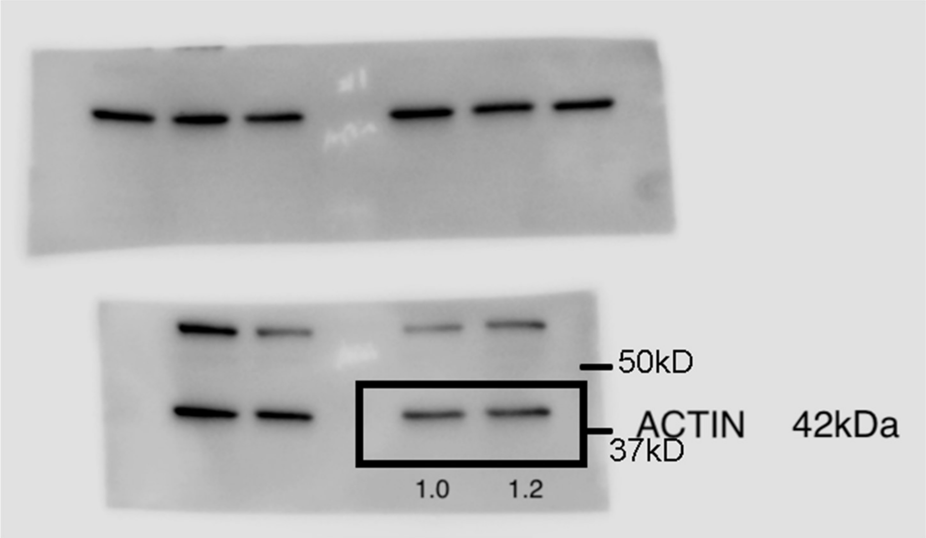

actin 1

Figure S7. Full Western Blot images for Figure S1E. Densitometry readings for each band: 410 shAR 1: 30300.14; 1163.92; actin-1: 43083.50; 50483.79.

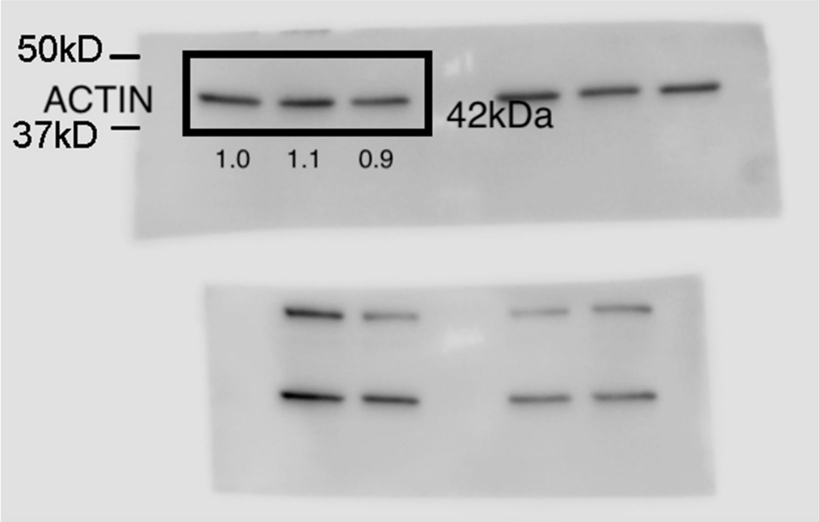

014 shJ shF-actin-5

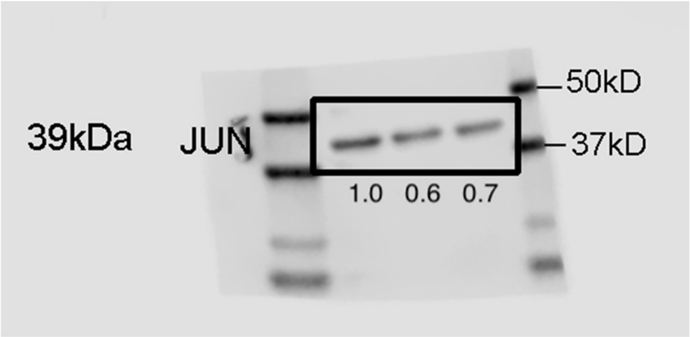

014shJ-J 1-1

Figure S8. Full Western Blot images for Figure S2A. Densitometry readings for each band: 014 shJ-J 1-1: 39490.55; 26858.55; 24935.70; 014 shJ shF-actin 5: 45492.36; 52127.74; 42466.55.

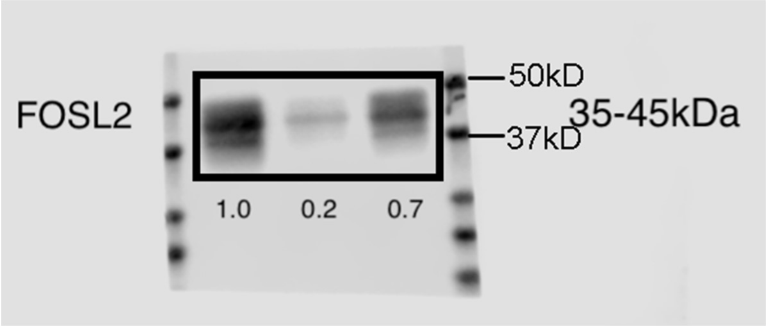

014 shf-2

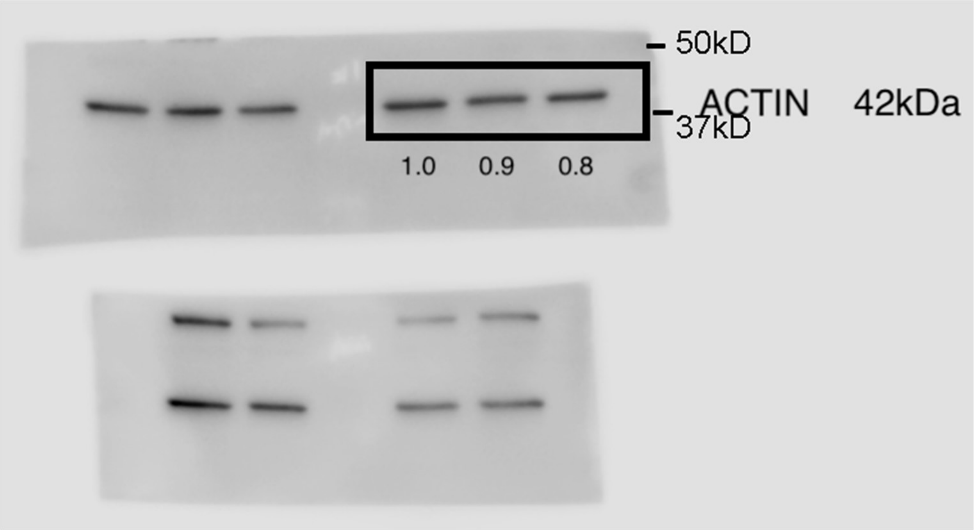

014 shJ shF-actin 5

Figure S9. Full Western Blot images for Figure S2C. Densitometry readings for each band: 014 shF-2: 49716.673; 7036.397; 28270.589; 014 shJ shF-actin 5: 52892.560; 47248.794; 40315.066.
